# Intravascular Large B-Cell Lymphoma Presenting as Interstitial Lung Disease

**DOI:** 10.1155/2014/928065

**Published:** 2014-05-15

**Authors:** Elham Vali Khojeini, Joo Young Song

**Affiliations:** ^1^Department of Pathology and Laboratory Medicine, University of California, Davis, 4400 V Street, Sacramento, CA 95817, USA; ^2^Department of Pathology, City of Hope, 1500 E Duarte Road, Duarte, CA 91010, USA

## Abstract

Intravascular large B-cell lymphoma (IVLBL) is a rare subtype of diffuse large B-cell lymphoma that resides in the lumen of blood vessels. Patients typically present with nonspecific findings, particularly bizarre neurologic symptoms, fever, and skin lesions. A woman presented with shortness of breath and a chest CT scan showed diffuse interstitial thickening and ground glass opacities suggestive of an interstitial lung disease. On physical exam she was noted to have splenomegaly. The patient died and at autopsy was found to have an IVLBL in her lungs as well as nearly all her organs that were sampled. Although rare, IVLBL should be included in the differential diagnosis of interstitial lung disease and this case underscores the importance of the continuation of autopsies.

## 1. Introduction

Intravascular large B-cell lymphoma (IVLBL) is a rare variant of diffuse large B-cell lymphoma that is characterized by the selective growth of lymphoma cells within the lumen of the vessels, particularly capillaries, with the exception of large arteries and veins [1]. The clinical presentation can be highly variable and often suggests infection rather than neoplasm. Clinical manifestations include constitutional symptoms, skin lesions, stroke, focal neurologic deficits, dyspnea, hepatosplenomegaly, and splenic infarction. The neurologic deficits are from the presence of vascular occlusions in the brain resulting in infarcts. Cutaneous manifestation is common but nonspecific. This type of presentation of IVLBL is exceedingly rare but should be considered in the differential diagnosis of interstitial lung disease.

## 2. Case Presentation

**Figure 1:**
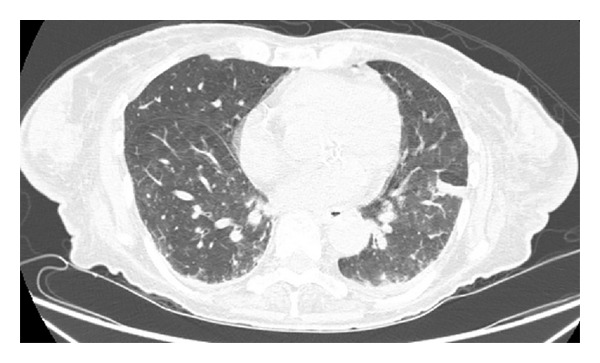
Computed tomography (CT) scan of the chest showed diffuse interstitial thickening and ground glass opacities.

A 76-year-old female with a past medical history of lumbar spine stenosis for over 5 years presented with 13.6 kg weight loss in the past nine months with no change in her diet and shortness of breath. A computed tomography (CT) scan of the chest showed diffuse interstitial thickening and ground glass opacities with a basilar predominance within the lungs but no honeycombing was seen (Figure 1). A pulmonary function test was performed and showed reduced diffusing capacity for carbon monoxide (DLCO < 40%). She was admitted to the hospital and placed on steroids as well as oxygen. During her hospital course she was found to have just progressive thrombocytopenia from 45,000/*μ*L to12,000/*μ*L without any other complete blood count abnormalities. Examination of the peripheral blood did not show any circulating atypical lymphocytes and a normal white blood cell differential. Also of note, the patient had abnormal liver function tests. A CT scan of the abdomen showed splenomegaly (18 × 12 × 10 cm) without adenopathy.

A bone marrow biopsy was performed because of the thrombocytopenia and splenomegaly which was reported as negative for a hematolymphoid neoplasm. Hemophagocytosis was not seen. She developed generalized edema and continued to decline. Eventually she became unresponsive and was pronounced dead. At autopsy she was found to have hepatosplenomegaly, massive hemoperitoneum (approximately 4 liters), and diffuse generalized soft tissue edema with bilateral chemosis.

**Figure 2:**
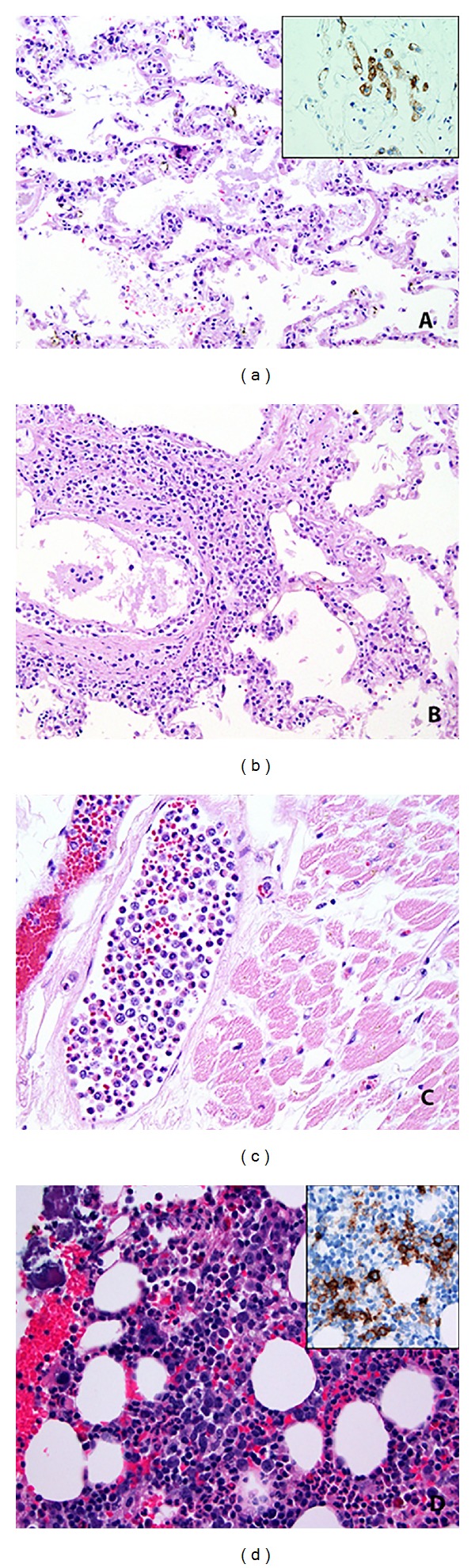
At autopsy, many of the organs showed involvement by intravascular diffuse large B-cell lymphoma. The alveolar wall showed large atypical cells in the capillaries (a) that were positive for CD20 (inset). Within the arteriole wall of the lung were these atypical cells as well (b). The myocardium also showed these large atypical cells in the vessel lumen (c). Retrospective evaluation of the bone marrow also showed inconspicuous large cells in clusters (d) highlighted with CD20 (inset).

On microscopic review of the tissue sections from the organs there was a diffuse infiltrate of large atypical cells with irregular nuclear contours, vesicular chromatin, and occasional prominent nucleoli. These cells were exclusively seen within the lumen of blood vessels of nearly all the organs sampled, including the liver, spleen, kidney, lungs (Figures 2(a) and 2(b)), heart (Figure 2(c)), aortic wall, and brain. The alveolar walls and capillaries of the lungs were infiltrated by these large atypical lymphocytes. Immunohistochemistry was performed and showed these large atypical cells were positive for CD20 (Figure 2(a) inset, Figure 2(d) inset), PAX-5, MUM1, and Bcl-2. Cytogenetics showed a normal karyotype. Based on these findings, the diagnosis was consistent with an intravascular large B-cell lymphoma (IVLBL).

## 3. Discussion

This case is interesting in that the patient presented with shortness of breath and had clinical findings consistent with interstitial lung disease (ILD), which is an unusual presentation for this type of lymphoma [2]. However, only at autopsy was the patient found to have involvement of IVLBL that clinically mimicked ILD. Recent studies have found that some cases have increased diffuse FDG-PET uptake in the lungs at diagnosis, which may help in alerting the clinician to add this disease in the differential [3, 4]. This case underscores the importance of the continuation of autopsies at medical centers as well as highlights an unusual presentation of IVLBL. Interestingly, reevaluation of the bone marrow biopsy (Figure 2(d)) showed the inconspicuous large B-cells within vessels only seen with immunohistochemistry.
